# Meetings of Societies

**Published:** 1911-12

**Authors:** 


### MEETINGS OF SOCIETIES.

### Bristol Medico-Chirurgical Society.

*Annual Meeting, October 11th,* 1911.

After a vote of thanks to the retiring President, Dr. Bertram M. H. Rogers, had been passed, Mr. C. A. Morton took the chair, and gave an introductory address (*see* p. 289). Later a cordial vote of thinks was given to Mr. Morton for his able and interesting address.

The Honorary Secretary and Editorial Secretary, Dr. J. A. Nixon, read the following annual report :—

There is a balance in hand on the General Account of £114 19s. 11d. From members' subscriptions the receipts were £182 14s. od. The balance from the previous year was £126 19s. 3d. The expenses amounted to £201 6s. 9d.

The *Journal* Account showed a deficit on the year 1910 of £17. This was met by a special grant from the Society's funds.

During the year one honorary member and twelve ordinary members were elected and thirteen resigned. We have to deplore the death of two members, Dr. John Beddoe, F.R.S., for many years an honorary member, and Dr. E. Blacker. On October 1st, 1911, there were 169 members.

The Hon. Librarian, Mr. C. K. Rudge, then read the following report :—

The Library of the Bristol Medico-Chirurgical Society now contains 11,619 volumes. During the year 231 volumes have been added. The Library is in regular receipt of 190 periodicals.

During the year 912 loans of books were made, as compared with 1,107 in the preceding year. The difference is accounted for by the borrowing of books being stopped during the removal. Fifty volumes had been obtained from Lewis's Library, the membership to which is found to be of great service to readers.

The Bristol Medical Library, to which members of the Society have access, now contains 21,929 volumes, made up in the following manner :—

BOOKS.

| | |
|---|---|
| Medico-Chirurgical Society .. .. | 11,619 |
| University of Bristol .. .. .. | 8,547 |
| Bristol Royal Infirmary .. .. | 1,189 |
| Bristol General Hospital .. .. | 574 |
| Total .. .. | 21,929 |

CURRENT PERIODICALS.

| | | |
|---|---|---|
| Medico-Chirurgical Society | .. .. | 190 |
| University of Bristol .. | .. .. | 59 |
| Total | .. .. | 249 |

The following are the officers of the Society for the Session 1911–12 : President—C. A. Morton, F.R.C.S. President-Elect —W. C. Swayne, M.D., B.S. Lond., M.D., Ch.B. Bristol. Committee (*ex officio*)—B. M. H. Rogers, B.A., M.D. Oxon. (retiring President) ; R. Shingleton Smith, M.D. Lond. (Editor of *Journal*) ; C. King Rudge, M.R.C.S. (Hon. Librarian) ; J. A. Nixon, B.A., M.B. Cantab. (Editorial Secretary). (*Elected*)— G. Munro Smith, M.R.C.S., and J. Michell Clarke, M.A., M.D. Cantab. (Ex-Presidents) ; P. Watson-Williams, M.D. Lond. ; G. Parker, M.A., M.D. Cantab. ; I. Walker Hall, M.D.Vict. ; F. Lace, F.R.C.S. ; J. Lacy Firth, M.D., M.S. Lond. ; John Wallace, M.B., C.M., B.Sc. Edin. Honorary Secretary—J. A. Nixon, B.A., M.B. Cantab.

**RECEIPT AND EXPENDITURE ACCOUNT OF THE BRISTOL MEDICO-CHIRURGICAL SOCIETY, SESSION 1910-11.**

| | £ | s. | d. | | £ | s. | d. |
|---|---|---|---|---|---|---|---|
| Balance in hand, Oct. 1st, 1910.. .. | 126 | 19 | 3 | Honorarium .. .. .. .. | 10 | 0 | 0 |
| Members' Subscriptions | 182 | 14 | 0 | Expenses of Capt. Mackie's Address .. | 7 | 15 | 1 |
| Interest on Deposit Account .. .. .. .. | 1 | 10 | 2 | Lantern at Meetings .. | 1 | 11 | 6 |
| Donation from Capt. Mackie, I.M.S. .. | 4 | 4 | 0 | Library Grant .. .. | 50 | 0 | 0 |
| Sundry Amounts.. .. | 0 | 19 | 3 | Rent, etc. .. .. .. | 57 | 6 | 0 |
| | | | | Porter .. .. .. .. .. | 1 | 2 | 0 |
| | | | | Printing .. .. .. | 3 | 9 | 0 |
| | | | | Postage .. .. .. .. .. | 4 | 1 | 2 |
| | | | | Editorial Secretary— Members' Subscriptions .. .. .. .. | 46 | 5 | 0 |
| | | | | Special Grant to *Journal* .. .. .. | 17 | 0 | 0 |
| | | | | Hon. Sec.'s Petty Cash | 2 | 17 | 0 |
| | | | | Balance in Bank .. .. | 114 | 19 | 11 |
| | £316 | 6 | 8 | | £316 | 6 | 8 |

---

*November 8th*, 1911.

Mr. C. A. Morton, President, in the Chair.

On the proposition of the President, seconded by Mr. Munro Smith, the following gentlemen were elected honorary members of the Society : R. Shingleton Smith, M.D., B.Sc., F.R.C.P., Consulting Physician to the Bristol Royal Infirmary, Emeritus Professor of Medicine in the late University College, Bristol ;

N. C. Dobson, F.R.C.S., Consulting Surgeon to the Bristol General Hospital, Emeritus Professor of Surgery in the late University College, Bristol; L. M. Griffiths, M.R.C.S. Eng., L.R.C.P. Edin., formerly Honorary Librarian to the Bristol Medical Library.

Dr. J. R. Charles showed a boy with **Amyotonia congenita.** Signs of the affection had been present at birth. The birth had been normal. The muscles of the limbs and to a less degree those of the trunk were remarkably atonic; partial dislocations of the wrists and knees were permitted; the feet were quite flat. The patient sat with kyphotic spinal curvature, though the spine could be easily straightened. The knee-jerks were good, the plantar reflexes flexor; the wrist and biceps jerk had only been obtained once or twice. There was no reaction of degeneration, but sluggish response to the interrupted current. The boy's intelligence was good.—Dr. Fortescue-Brickdale discussed the differences between this affection and amyotrophic affections. In the latter the trunk muscles became more affected, interfering with respiration, and the prognosis was worse. The speaker thought the cases of amyotonia in which spinal cord changes had been described were not true examples of the affection.—Dr. Carey Coombs alluded to an example of the disease he had shown in 1905, and described in the *British Medical Journal.* In his case the face had been affected in the late stages. It was conceivable that some of the spinal changes described as occurring might be secondary to the muscular atrophy.—Dr. Charles, in replying, referred to the fact that there might or might not be spinal cord changes following amputation of a limb. It was difficult to determine whether the spinal cord changes in amyotonia were primary or secondary.

Mr. A. L. Flemming read notes on the following cases:—(1) **Hypernephroma of the liver** (patient shown). The patient, a young woman, began to fail in health four years ago. She became feeble, with headaches, lassitude and amenorrhœa. The skin became bronzed, and there was marked growth of hair on the cheeks, chin and limbs, but loss of hair on the scalp. All these changes had disappeared since the removal last July of a hypernephroma of the liver. At the date of operation the patient was suffering from jaundice and attacks of colic, and the abdomen was explored by Mr. Carwardine. Two nodules were found on the liver in such a position that they pressed upon the common bile-duct. They had a diameter of about a quarter of an inch. The structure after removal was found to be that of hypernephroma. The skin discolouration had nearly disappeared since; but the patient was losing weight and weakness was increasing. (2) **Tumour of the cheek treated**

**by radium** (patient shown). The patient had had a small tumour in the inside of the right cheek since seven years of age. Between June and October, 1910, it doubled in size, and was therefore removed by Mr. Walters, and found to be an endothelioma. After a month the cheek at the seat of operation became painful, and a tumour formed again, and rapidly, within a few days, reached a diameter of 1½ to 2 inches. An exploratory incision, which evacuated a drop or two of pus, did no good, and the tumour continued to enlarge. A section of a portion of mucous membrane showed endotheliomatous growth. In a few days the whole cheek became involved, and treatment by radium was decided upon. Four applications were made by Dr. Finzi, and the tumour disappeared, leaving a supple scar. (3) **Filaria loa.** An adult filaria loa removed from beneath the conjunctiva of a patient was shown. The patient, who had been previously treated for the disease, and was known to still have the parasite in the blood, was asked to come to see the speaker immediately if any eye symptoms developed. He appeared one morning at seven, and the worm was seen moving beneath the conjunctiva. It was removed with Dr. Stack's help.—Mr. Walters commented upon the tumour of the cheek. When the incision was made to explore the recurrent swelling the tissues cut like carcinoma. Radium first localised the swelling remarkably and then caused its disappearance.—Dr. James Swain, who had also seen the case, commented upon the remarkable effects of the radium. He pointed out that endotheliomata varied much in malignancy, and he thought they were probably more amenable to radium than carcinomatous tumours ; also he considered recurrent tumours were more amenable than primary ones. He described a case of malignant glandular tumour of the neck which had recurred after operation and been treated with radium by Dr. Wickham. The tumour disappeared for a time, but ultimately recurred and caused death.—Dr. J. O. Symes alluded to a case of rodent ulcer successfully treated by radium.—Dr. Watson-Williams described a case of œsophageal carcinoma in which great relief of dysphagia was twice produced by radium application by Dr. Finzi, but ultimately the symptoms returned and further treatment became impossible.

Dr. Watson Williams read a note on **Cardio-vascular nasal reflex influences.** A series of lantern slides displaying a section of the medulla of a new-born mouse, by Cajal, and various diagrams showed the connections between the substantia gelatinosa and the vagal efferent nucleus, thus explaining the influence of the sensory fifth nerve in the nose on the territory of the vagus. Further slides were shown to draw attention to experimental proof of the influence of the olfactory and fifth nerves on the pulse.

Dr. S. V. STOCK, commenting on the paper, said he had noticed that separation of the mucous membrane from the nasal septum in the operation of sub-mucous resection caused a fall in blood-pressure. Removal of the bony crest at the base did not have this effect ; also, if the operation was done under local anæsthesia with 20 per cent. cocaine, there was not a fall in blood-pressure.—Dr. CAREY COOMBS alluded to a case of tachycardia, in which the attacks were ushered in with nasal symptoms like those of hay-asthma.

Dr. NEWMAN NEILD read clinical notes on **Tea-poisoning.** (See page 312.)

J. LACY FIRTH.
J. A. NIXON, *Hon. Sec.*

